# Supplementary material for: Lamin A‐mediated nuclear lamina integrity is required for proper ciliogenesis
Source: EMBO Rep. 2020 Aug 19;21(10):e49680. doi: 10.15252/embr.201949680 (PMC7534621; doi:10.15252/embr.201949680)
Supplement: Supplementary file 1 — Appendix [file EMBR-21-e49680-s001.pdf]

Appendix for

Lamin A-mediated nuclear lamina integrity is required for proper ciliogenesis

Jia-Rong Fan, Li-Ru You, Won-Jing Wang, Wei-Syun Huang, Ching-Tung Chu, Ya-Hui Chi, and Hong-Chen Chen*

*Corresponding author. Email: hcchen1029@ym.edu.tw

**Content**	**Page**
Appendix Figure S1	2
Appendix Figure S2	3
Appendix Figure S3	4
Appendix Figure S4	5
Appendix Figure S5	6
Appendix Figure S6	7
Appendix Figure S7	8
Appendix Figure S8	9
Appendix Figure S9	10
Appendix Figure S10	10
Appendix Figure S11	11
Appendix Figure S12	12
Appendix Figure S13	13
Appendix Figure S14	14
Appendix Figure S15	14

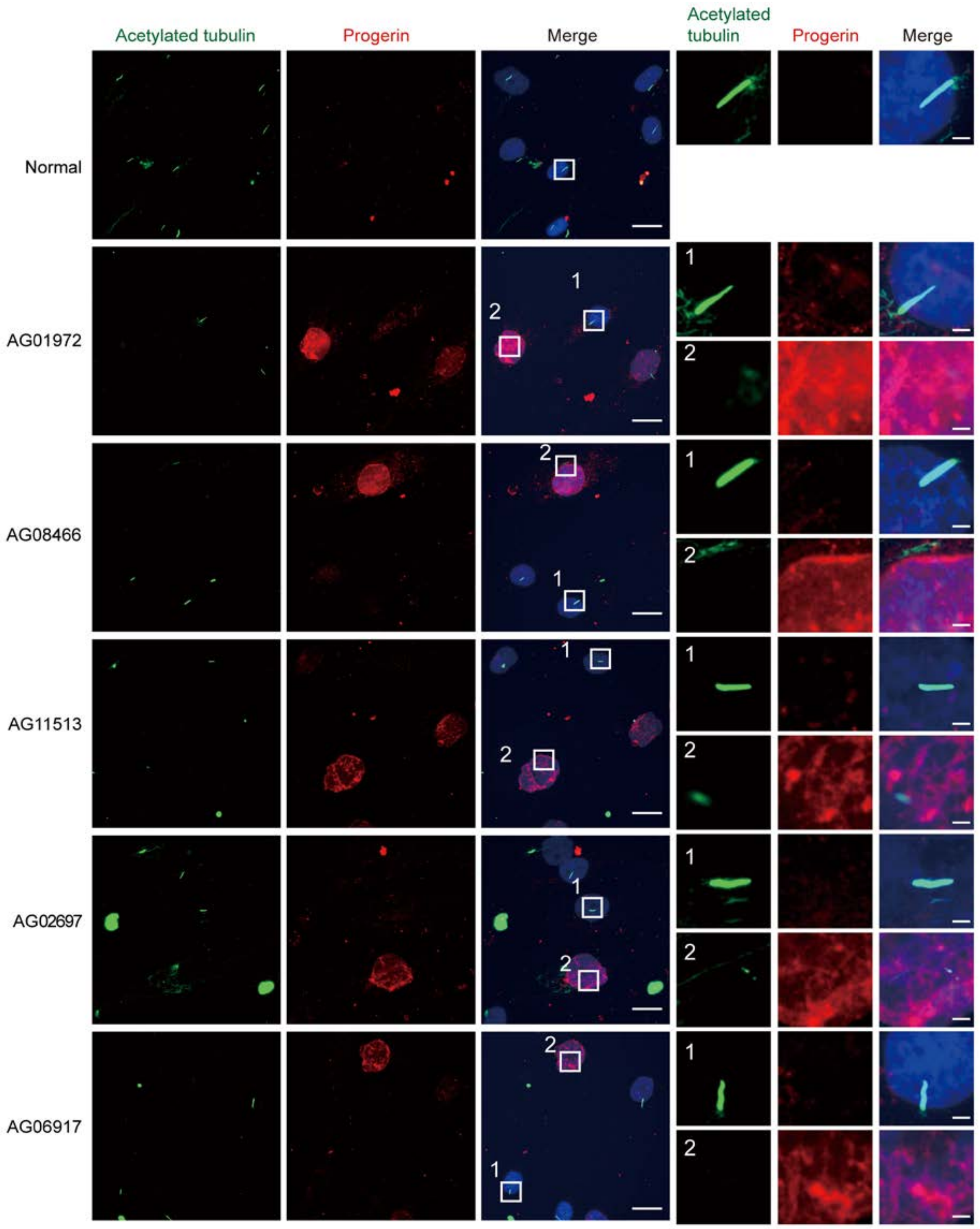

Appendix Figure S1. High progerin-expressing HGPS fibroblasts display defective ciliogenesis. Normal human fibroblasts and HGPS fibroblasts were serum-starved for 48 h and stained for progerin (red), acetylated tubulin (green) and DNA (blue). The representative images show that the cell with high progerin (inset 2), but not low progerin (inset 1), displays defective cilia formation. Scale bars, 20 μm or 2 μm (magnified images).

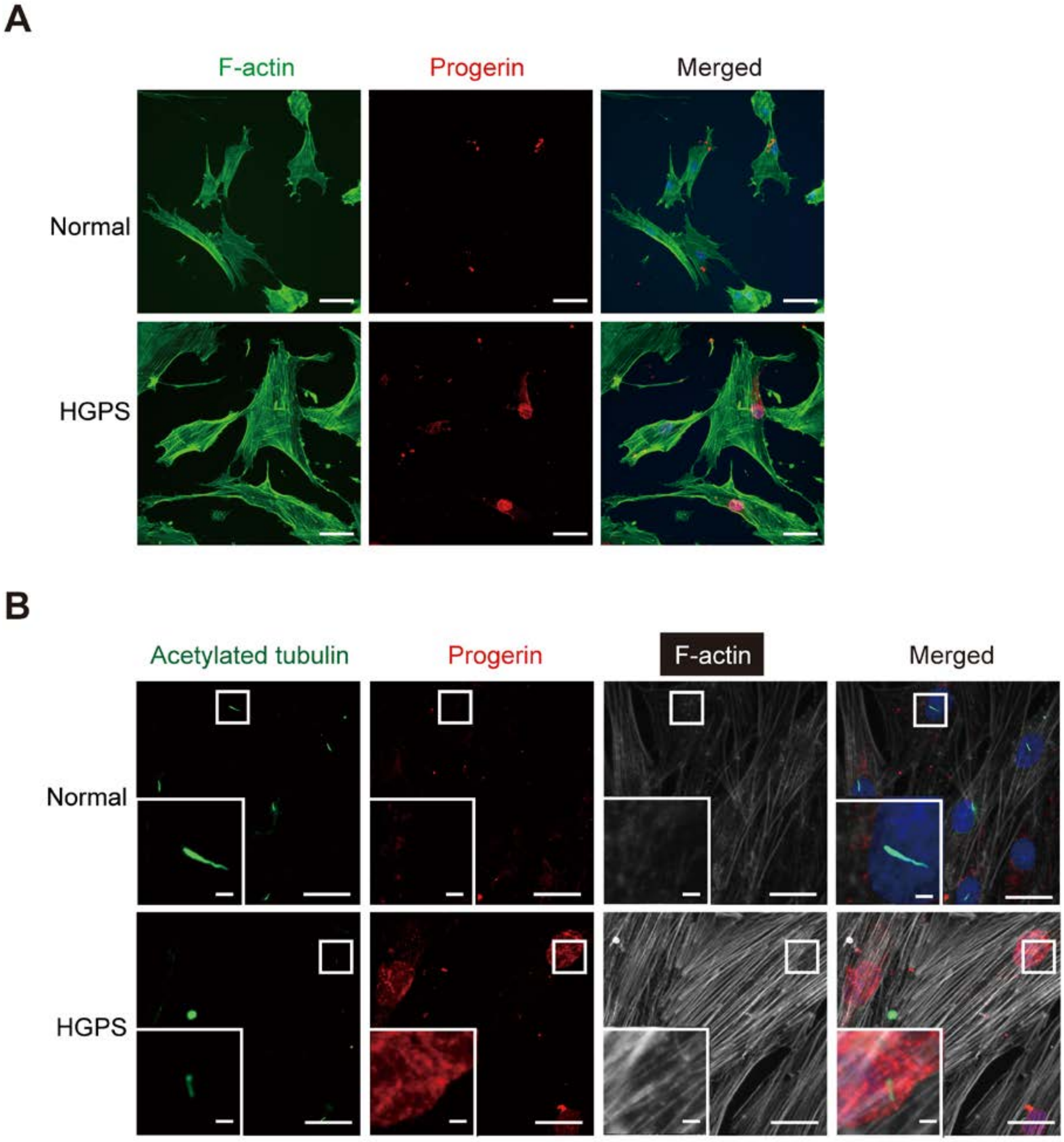

Appendix Figure S2. Increased cell spreading and prominent actin filaments in HGPS fibroblasts. (**A**) Normal human fibroblasts and HGPS fibroblasts were grown in the growth medium and stained for progerin (red) and F-actin (green). Scale bars, 50 µm. (**B**) Cells were serum-starved for 48 h and stained for progerin (red), acetylated tubulin (green), F-actin (white) and DNA (blue). Scale bars, 20 µm or 2 µm (magnified insets).

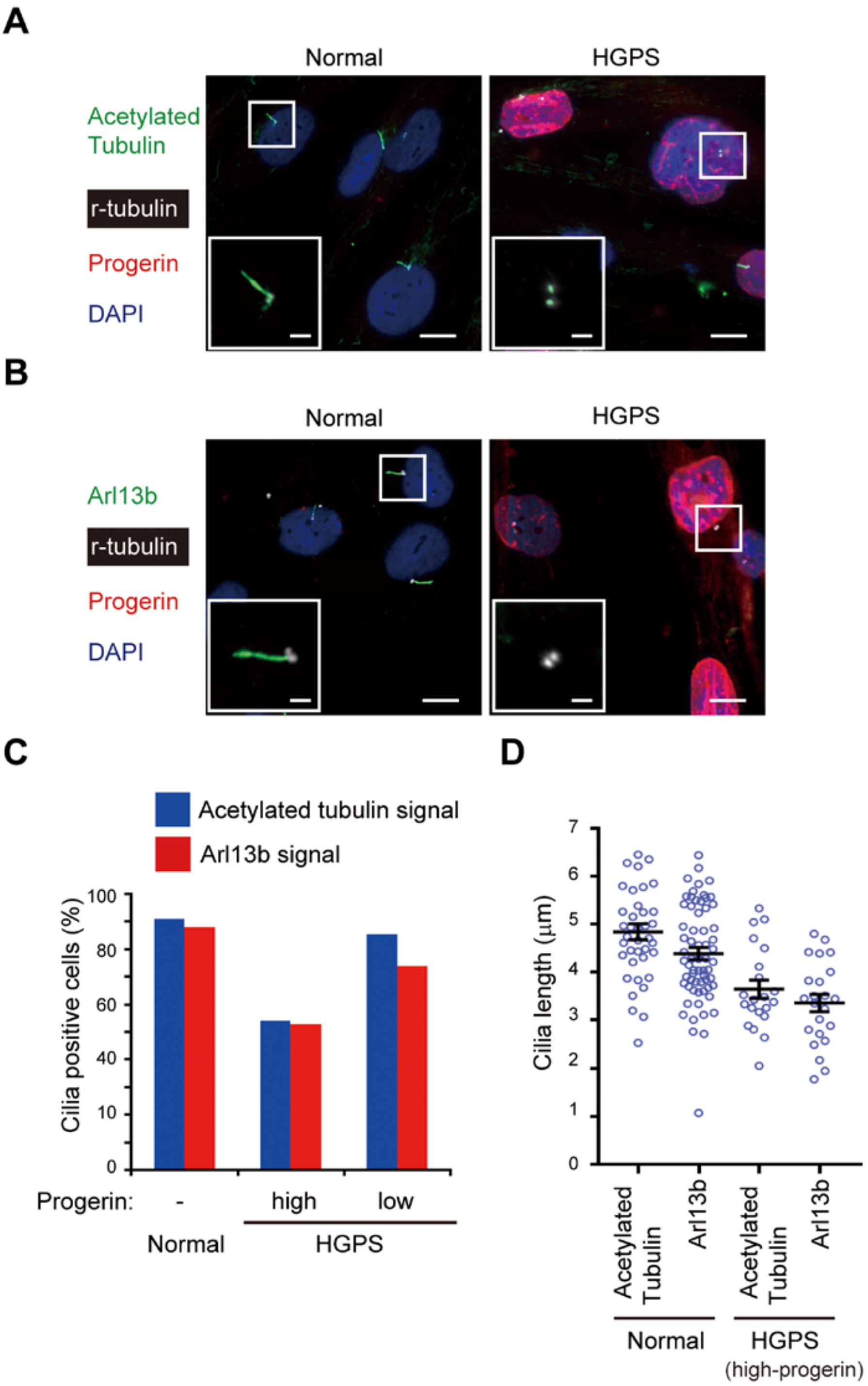

Appendix Figure S3. Confirmation of cilia deficiency in HGPS fibroblasts by the axonemal marker Arl13b. (A,B) Normal human fibroblasts and HGPS fibroblasts were serum-starved for 48 h and stained for progerin (red), γ-tubulin (white), DNA (blue), acetylated tubulin (green in A) or Arl13b (green in B). Scale bars, 10 μm or 2 μm (magnified insets). **(**C,D) The percentage of the cells with cilia in the total counted cells (C, n ≥ 50) and the cilia length (D, n ≥ 50) were measured according to the signals of acetylated tubulin or Arl13b. Values in C and D are from one experiment. Error bars in D, means ± s.e.m.

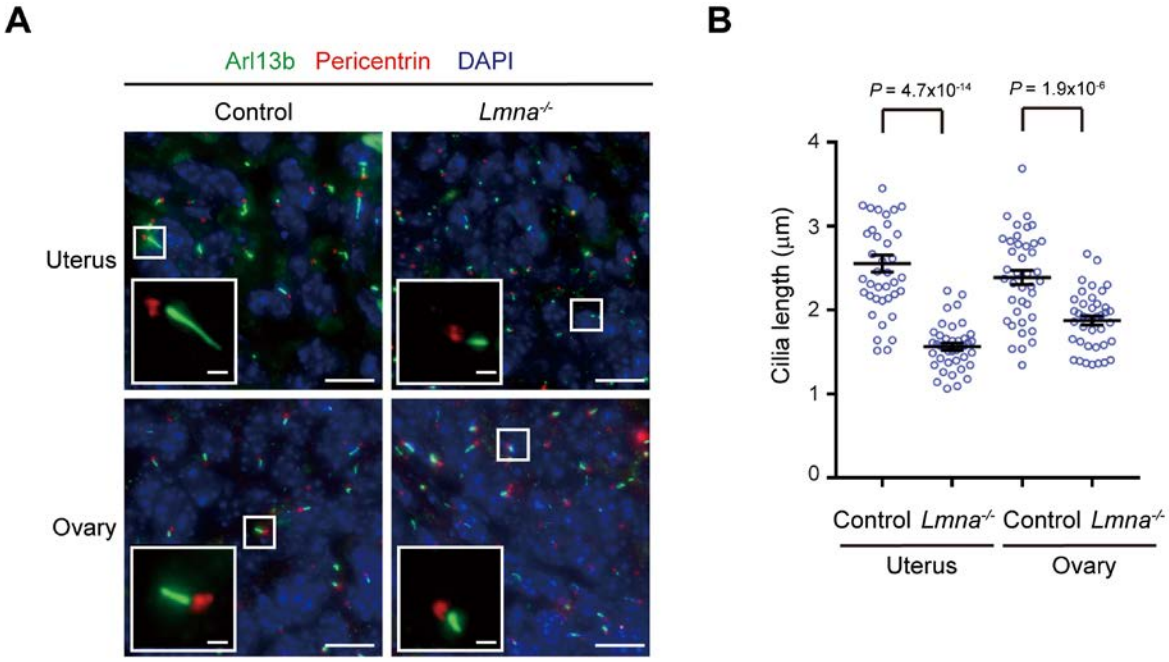

Appendix Figure S4. Confirmation of cilia deficiency in the uterus and ovary of *Lmna* null mice by the axonemal marker Arl13b. (A) The uterus and ovary from control and *Lmna*$^{-/-}$ mice were stained for Arl13b (green) and pericentrin (red). Scale bars, 10 µm or 1 µm (magnified insets). **(B)** The cilia length (n=40) in each organ from 4-week-old *Lmna*$^{-/-}$ mice (N=2) and their littermate controls (N=2) were measured. Values (means ± s.d.) are shown. Statistical significance of differences is assessed with a Student's t-test.

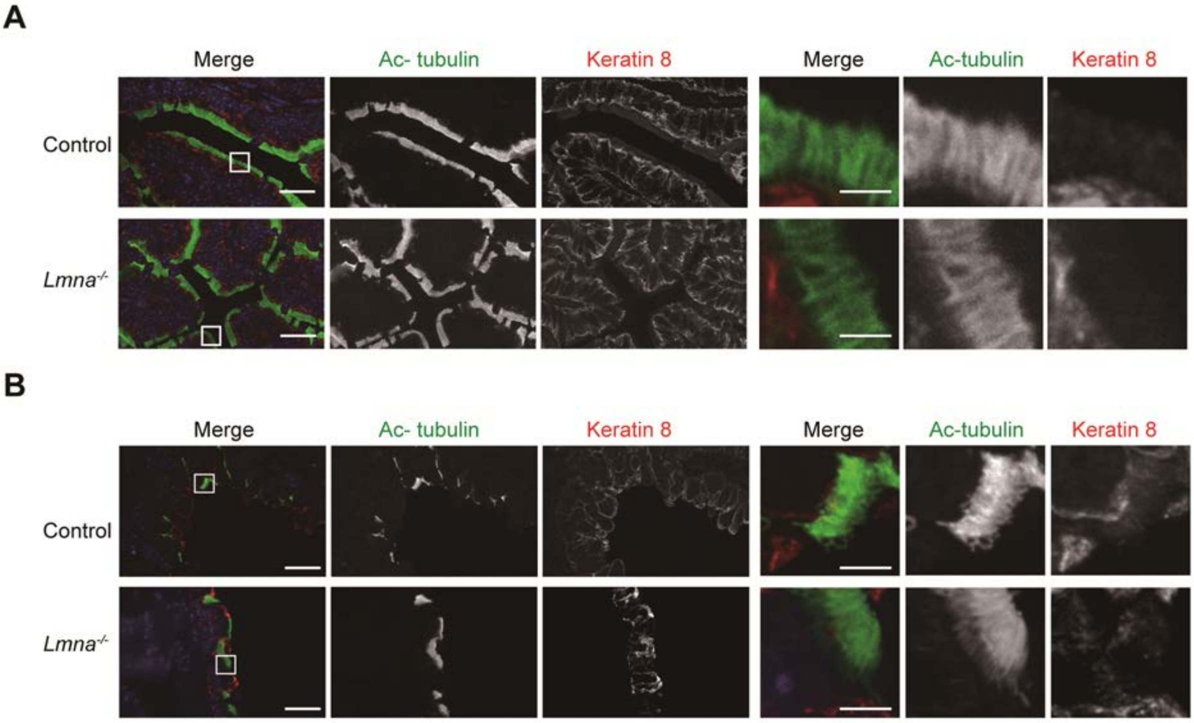

Appendix Figure S5. The motile cilia appear normal in *Lmna*$^{-/-}$ mice. The oviduct (**A**) and bronchus (**B**) of control and *Lmna*$^{-/-}$ mice were stained for acetylated tubulin (Ac-tubulin, green), keratin 8 (red) and DAPI (blue). The motile cilia were enlarged from the region indicated by the white box (in the main image) and shown in the magnified images. Scale bars, 20 μm (main image) or 2 μm (magnified images).

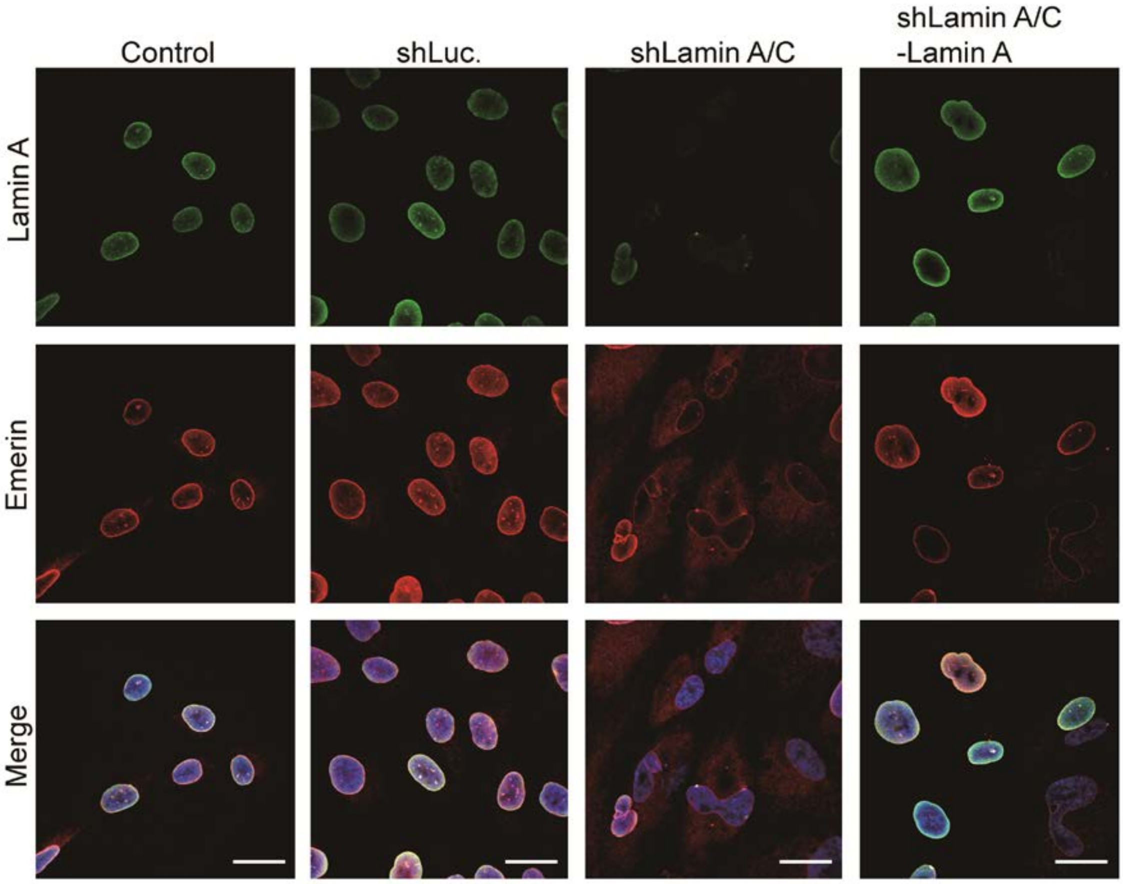

Appendix Figure S6. The depletion of lamin A/C causes curved nucleus and abnormal cytoplasmic distribution of emerin in RPE cells. RPE cells were infected with lentiviruses encoding shRNAs specific to lamin A/C (shLamin A/C) or luciferase (shLuc). Exogenous lamin A was re-expressed in the lamin A/C-depleted cells (shLamin A/C-Lamin A). The cells were stained for lamin A (green), emerin (red) and DNA (blue). Scale bars, 20 μm.

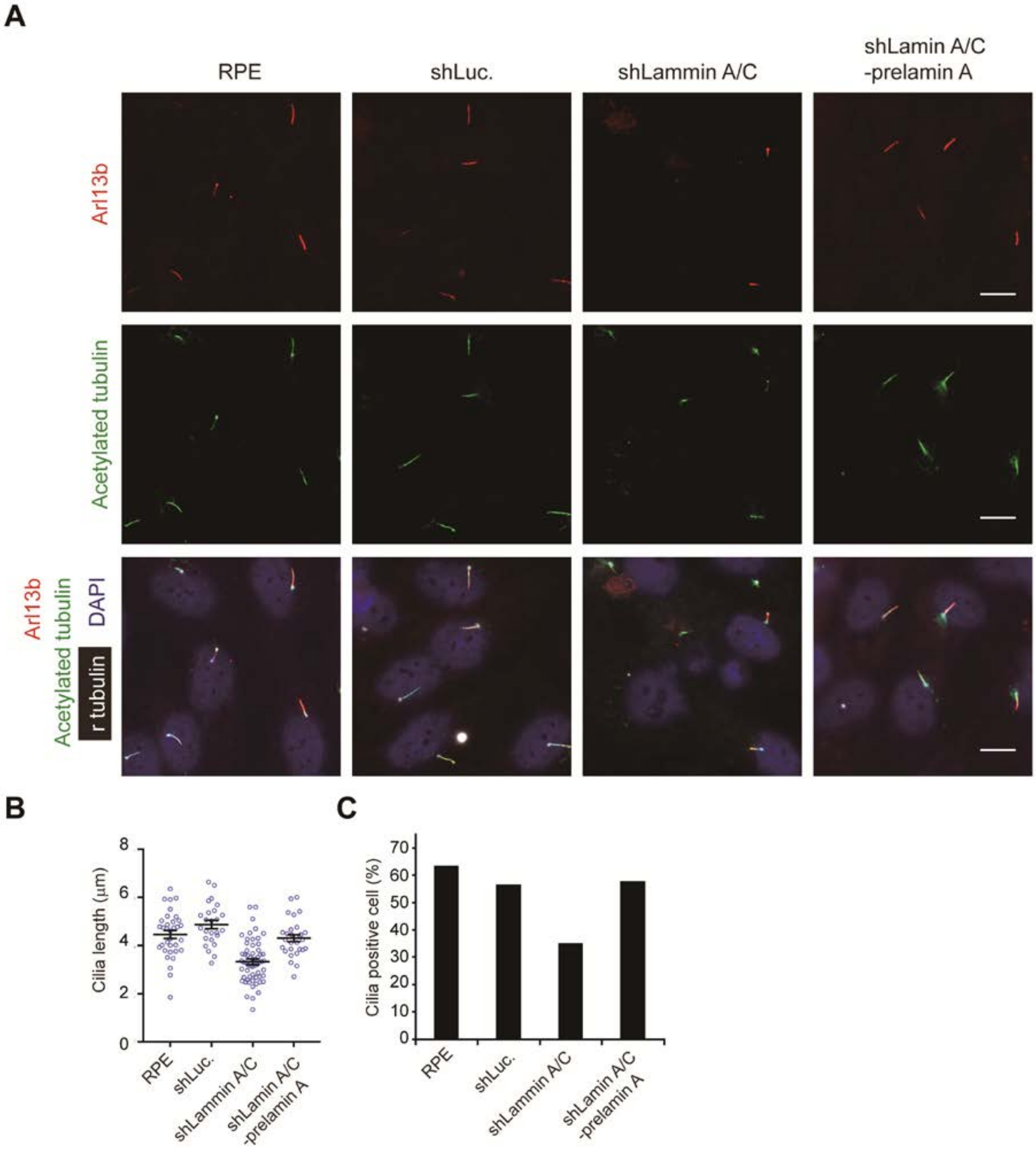

Appendix Figure S7. Confirmation of the ciliary deficiency by immunofluorescence stain with anti-Arl13b. (A) RPE cells were infected with lentiviruses encoding shRNAs specific to lamin A/C (shLamin A/C) or luciferase (shLuc). Exogenous lamin A was re-expressed in the lamin A/C-depleted cells (shLamin A/C-Lamin A). The cells were serum-starved for 48 h and stained for Arl13b (red), acetylated tubulin (green), γ-tubulin (white) and DNA (blue). Scale bars, 10 μm. **(B,C**) The length of cilia (B, $n \geq 30$ for each group) and cilia positive cells (C, $n \geq 57$ for each group) according to the signal of Arl13b were measured. Values in B and C are from one experiment. Error bars in B, means ± s.e.m.

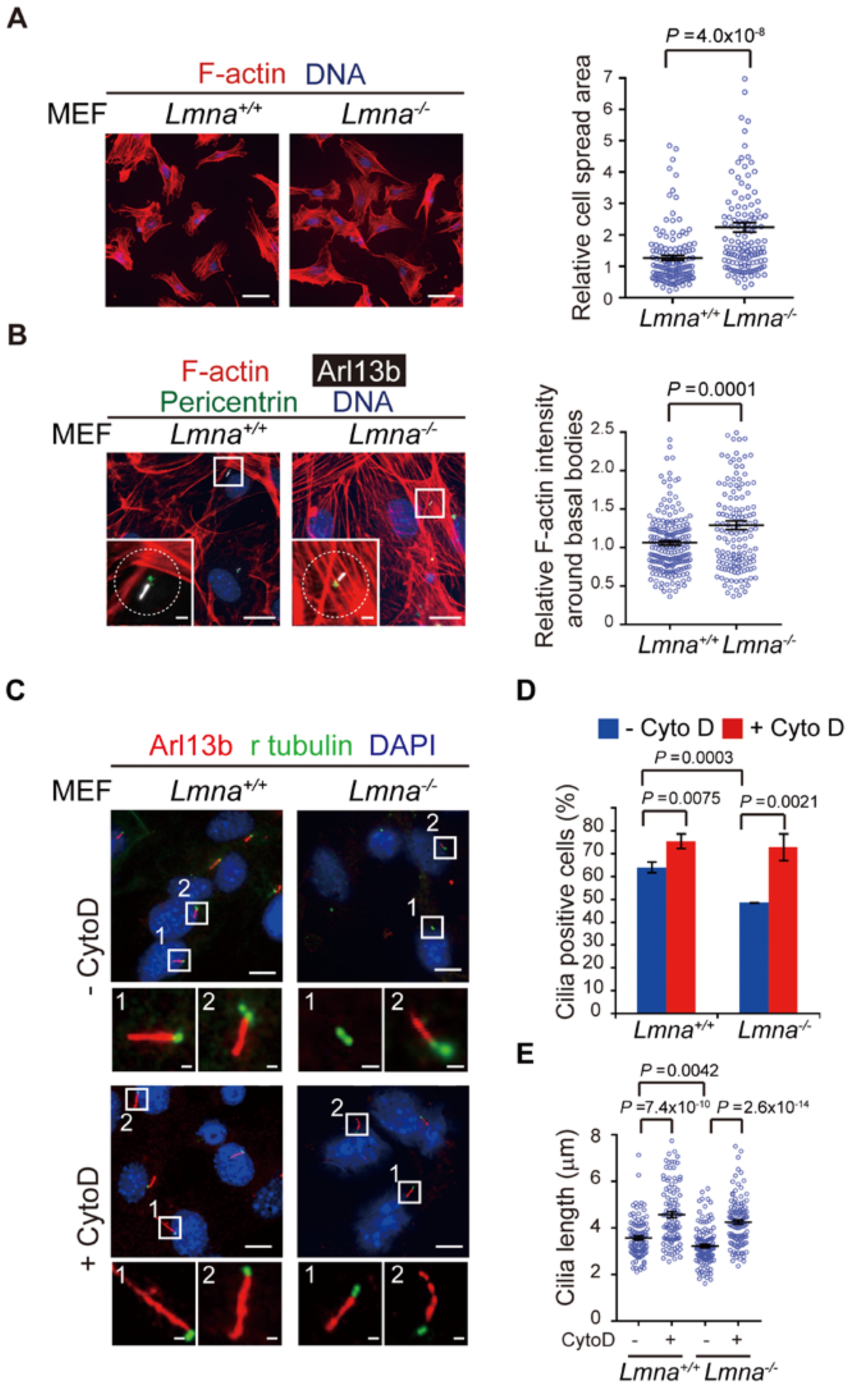

Appendix Figure S8. *Lmna*$^{-/-}$ MEFs display increased F-actin and defective primary cilia, both of which are rescued by Cyto D treatment. (A) The MEFs (*Lmna*$^{+/+}$ vs *Lmna*$^{-/-}$) were grown in the growth medium and stained for F-actin (red). The relative cell spreading area was measured according to F-actin staining (n ≥ 128). Scale bars, 50 μm. **(B)** *Lmna*$^{+/+}$ and *Lmna*$^{-/-}$ MEFs were serum-starved for 48 h and stained for F-actin (red), pericentrin (green) and Arl13b (white). The relative F-actin intensity within 100 μm^2 around the cilia (as illustrated by the circle) was measured (n ≥ 145 cells). Scale bars, 20 μm or 2 μm (in insets). **(C-E)** *Lmna*$^{+/+}$ and *Lmna*$^{-/-}$ MEFs were serum-starved for 32 h and treated with (+) or without (-) Cyto D (250 nM) for another 16 h. The cells were stained for Arl13b (red) and γ tubulin (green). The representative images are shown (C). Scale bars, 10 μm or 1 μm (magnified images). The percentage of the cells with cilia (D, n ≥ 288) and the length of cilia (E, n ≥ 97) were measured. DNA was stained by DAPI (blue, in A-C). Data information: Values (means ± s.e.m.) are from three independent experiments in A and E, and from two independent experiments in B. In D, values (means ± s.d.) are from three independent experiments. Statistical significance is assessed with a Student's t-test.

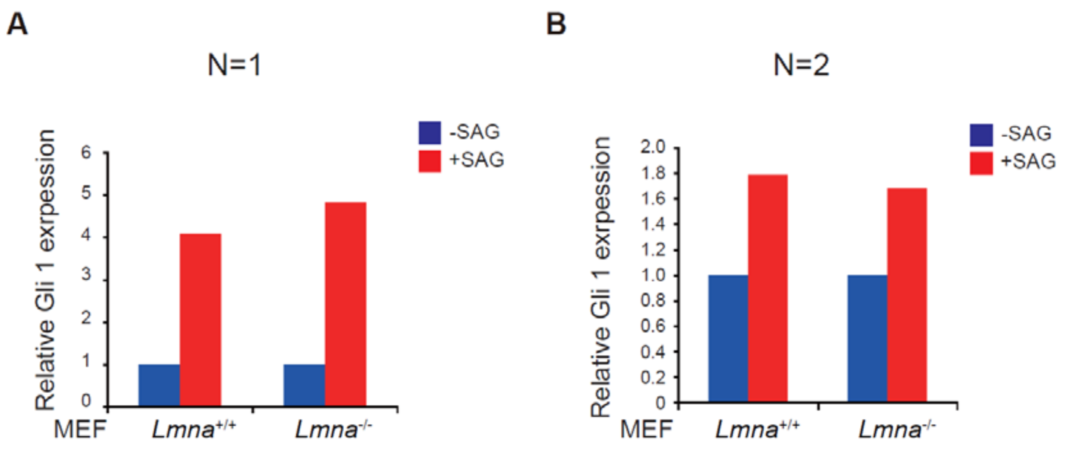

Appendix Figure S9. The Shh signaling is not affected in *Lmna*$^{-/-}$ MEFs. (**A-B**) *Lmna*$^{+/+}$ and *Lmna*$^{-/-}$ MEFs were serum-starved for 32 h and treated with SAG (400 nM) for 24 h. The relative Gli1 expression was then measured by quantitative RT-PCR. The results of two independent experiments are shown in A and B, respectively.

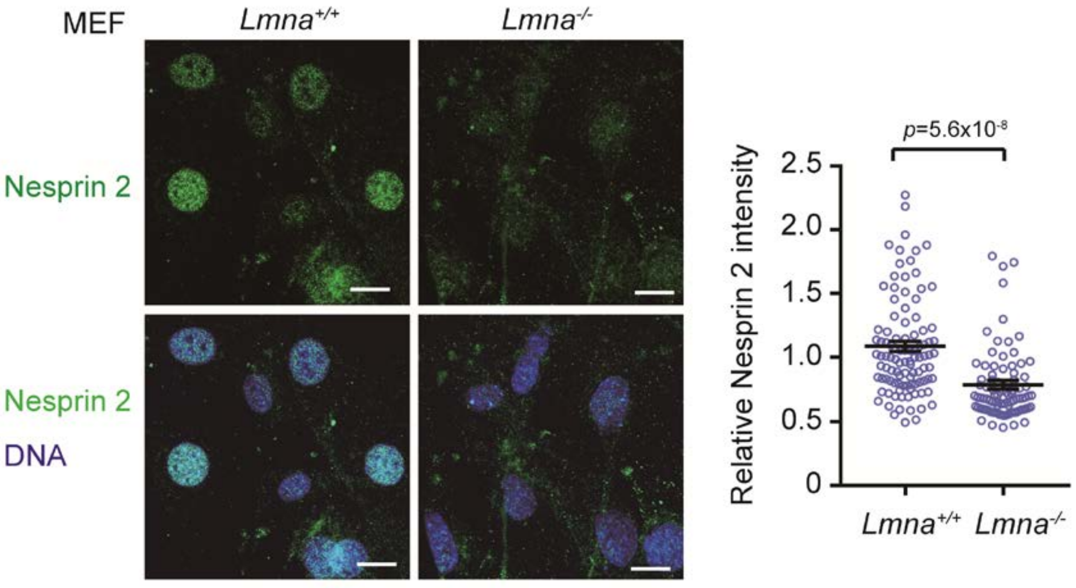

Appendix Figure S10. Downregulation of nesprin 2 in *Lmna*$^{-/-}$ MEFs. *Lmna*$^{+/+}$ MEFs and *Lmna*$^{-/-}$ MEFs were stained for nesprin 2 (green) and DNA (blue). The nesprin 2 fluorescence intensity of the cells was measured by the Zeiss ZEN2 software (n ≥ 91). Values (means ± s.e.m.) are from three independent experiments. Statistical significance of differences is assessed with a Student's t-test. Scale bars, 20 μm.

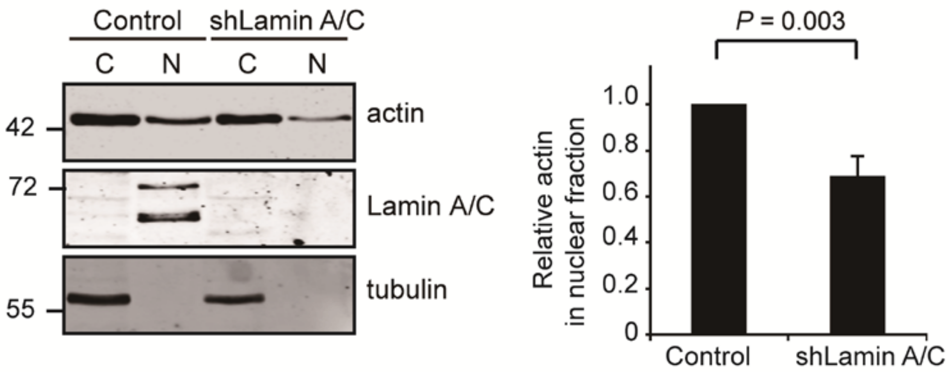

Appendix Figure S11. Actin level is reduced in the nuclear fraction of RPE cells upon lamin A/C depletion. An equal proportion of the lysates from the nuclear and cytoplasmic fractions of the control RPE cells and those expressing shRNAs to lamin A/C. The graph shows the quantification of relative amounts of actin in the nuclear fraction. Values (means ± s.d.) are from three independent experiments. Statistical significance of differences is assessed with a Student's t-test.

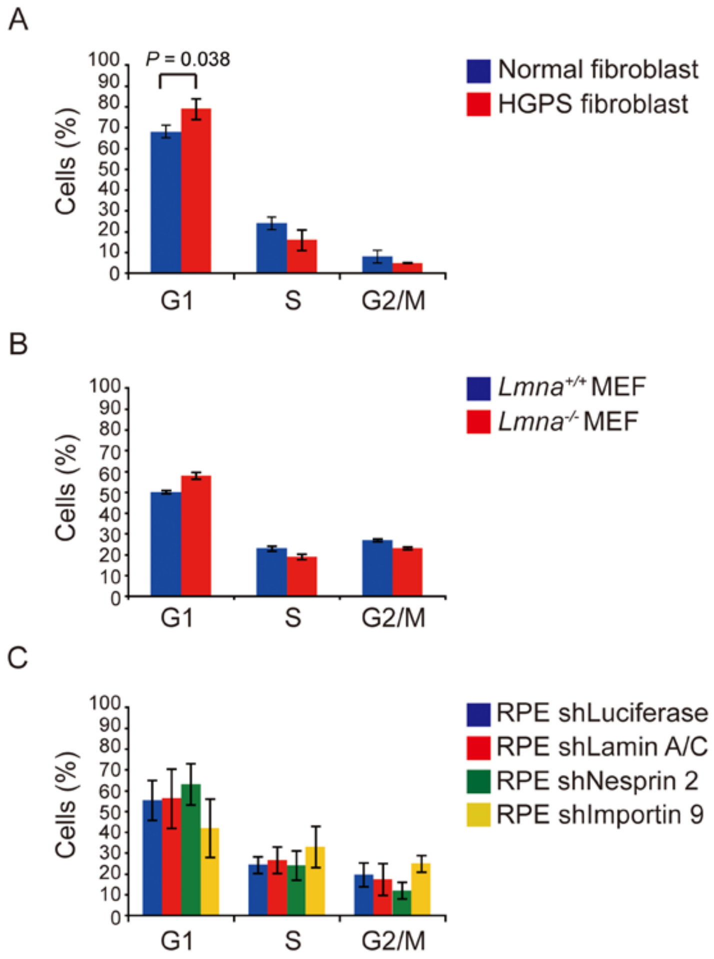

Appendix Figure S12. The cell cycle is not apparently altered in HGPS fibroblasts, *Lmna*$^{-/-}$ MEFs or by the different shRNA-treatments in RPE cells. (A-C) The cell cycle of fibroblasts (A, normal *vs* HGPS), MEFs (B, *Lmna*$^{+/+}$ *vs Lmna*$^{-/-}$) and RPE shLuciferase, shLamin A/C, shNesprin 2, shImportin 9 cells (in C) were analyzed by flow cytometry. The percentage of cells in G1, S, G2/M phases are shown. Values (means ± s.d.) are from three independent experiments in A, C, and from two independent experiments in B.

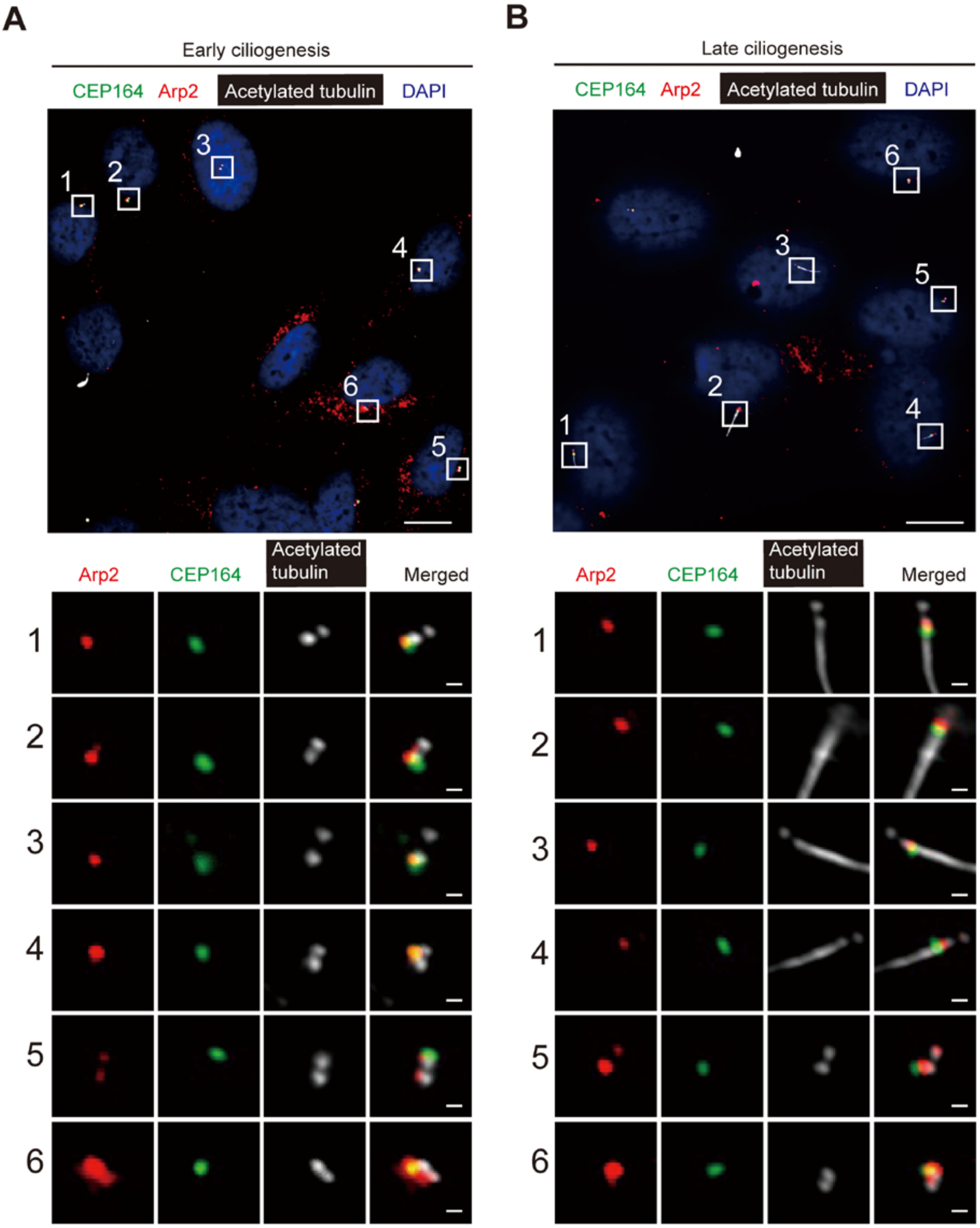

Appendix Figure S13. Arp2 localizes at the basal body during ciliogenesis. (**A,B**) RPE cells were serum-starved for 1 h (in A, as early ciliogenesis) or 48 h (in B, as late ciliogenesis) and then stained for Arp2 (red), CEP164 (green), acetylated tubulin (white) and DNA (blue). The low power images with six zoomed insets are shown. Note that Arp2 was found more frequently to localize exclusively at the mother centriole (insets 1-4) than at both mother and daughter centrioles (insets 5 and 6). Scale bars, 10 µm or 0.5 µm (magnified images).

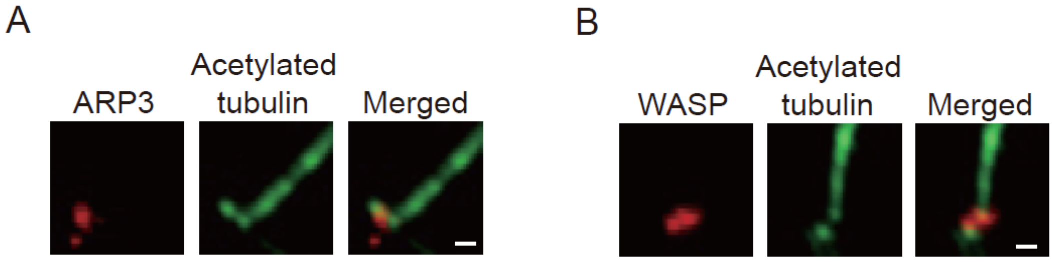

Appendix Figure S14. Arp3 and WASP localize to the centrosomes in ciliated RPE cells. (A-B) RPE cells were serum-starved for 48 h and then stained for acetylated tubulin (green), Arp3 (in A, red) or WASP (in B, red). The representative images are shown. Scale bars, 0.5 μm.

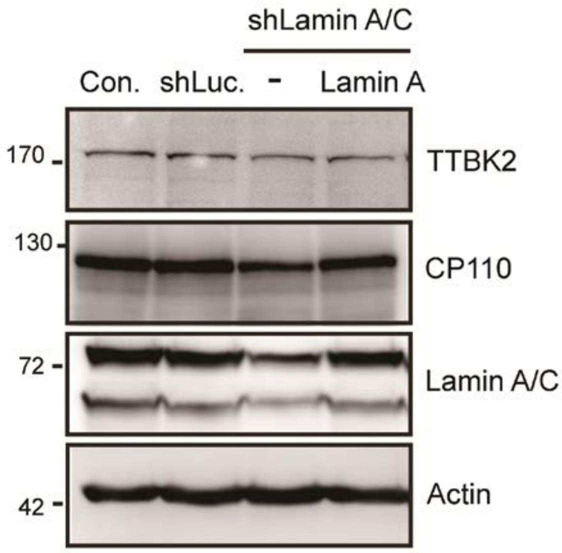

Appendix Figure S15. The expression levels of TTBK2 and CP110 are not affected by the depletion of lamin A/C. RPE cells were infected with lentiviruses encoding shRNAs specific to luciferase (shLuc.) or lamin A/C (shLamin A/C). Exogenous lamin A was re-expressed in the lamin A/C-depleted cells (shLamin A/C-Lamin A). An equal amount of whole cell lysates was analyzed by immunoblotting with antibodies as indicated.
