# Supplementary material for: Lamin A‐mediated nuclear lamina integrity is required for proper ciliogenesis
Source: EMBO Rep. 2020 Aug 19;21(10):e49680. doi: 10.15252/embr.201949680 (PMC7534621; doi:10.15252/embr.201949680)
Supplement: Supplementary file 2 — Expanded View Figures PDF [file EMBR-21-e49680-s002.pdf]

Expanded View Figures

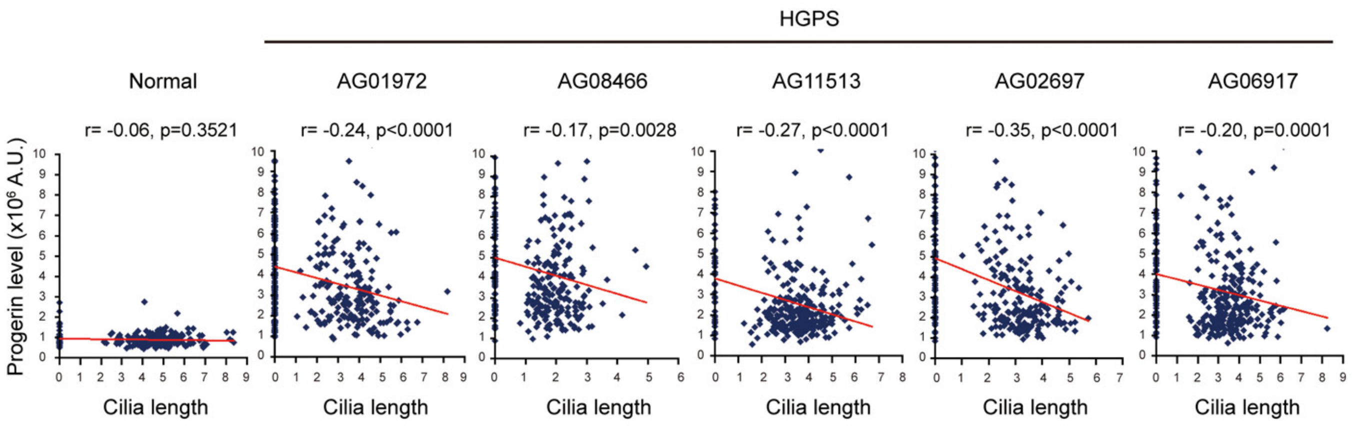

Figure EV1. An inverse correlation between progerin level and cilia length in HGPS fibroblasts.

Normal human fibroblasts and HGPS fibroblasts were serum-starved for 48 h and stained for progerin, acetylated tubulin and DNA. The progerin fluorescence intensity and cilia length of the cells were measured by the Zeiss ZEN2 software ($n \geq 225$). Pearson r and P values (Student's t-test) were determined in the correlation analysis by the Prism software.

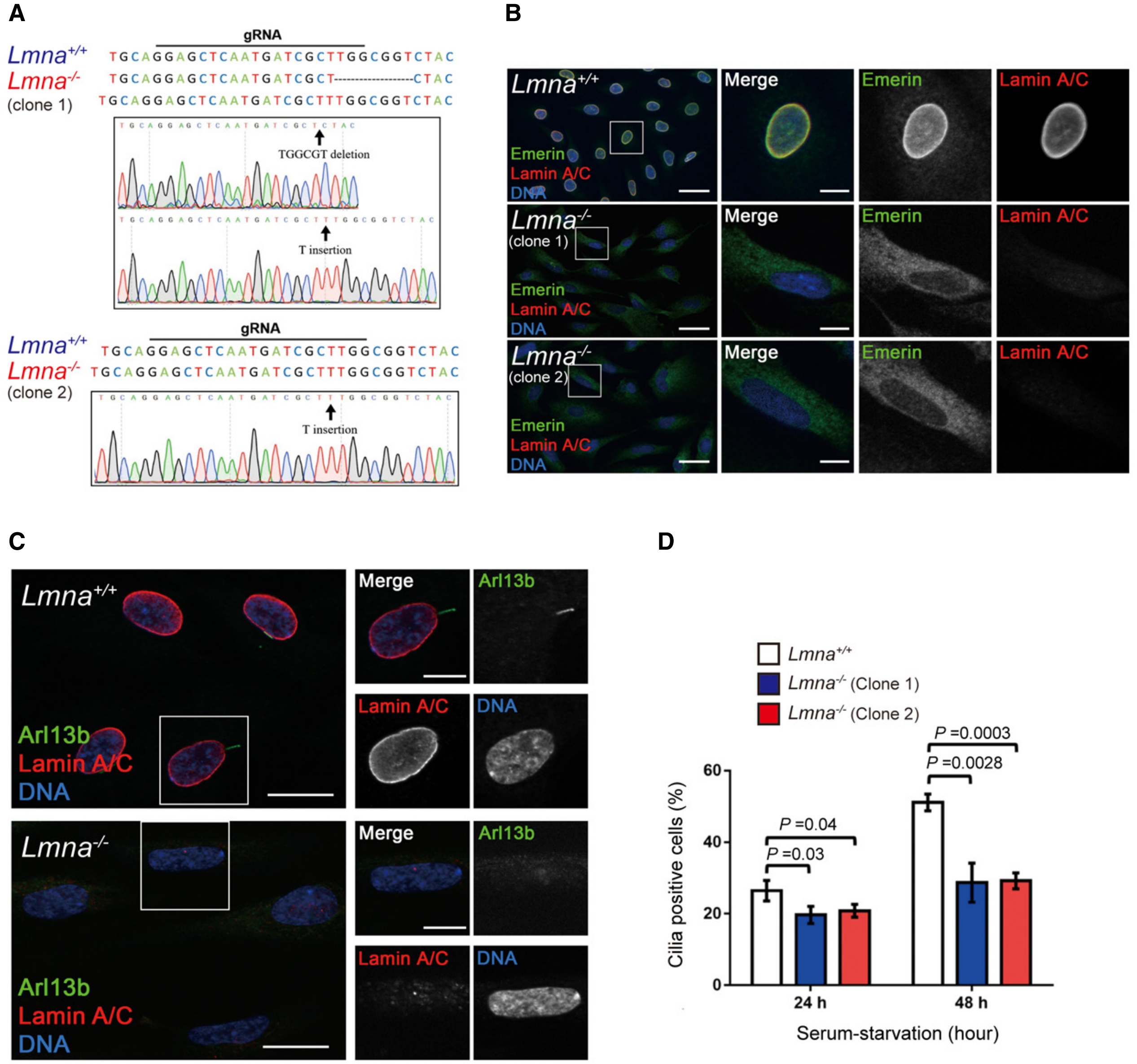

Figure EV2. Generation of *Lmna*$^{-/-}$ RPE cell lines, which show defective ciliogenesis.

A Genotyping of *Lmna*$^{-/-}$ RPE clone 1 (top) and clone 2 (bottom). In *Lmna*$^{-/-}$ RPE clone 1, two different mutations were identified within the gRNA targeting site, including a 7-nucleotide deletion (TGGCGGT) and a T insertion. The arrow indicates the deletion or insertion site. In RPE *Lmna*$^{-/-}$ clone 2, the same T insertion on both alleles was identified within the gRNA targeting site. The arrow indicates the insertion site.

B Abnormal cytoplasmic distribution of emerin in *Lmna*$^{-/-}$ RPE cells (clones 1 and 2). The cells were stained for lamin A/C (red) and emerin (green). Scale bars, 40 or 10 µm (magnified images).

C The *Lmna*$^{+/+}$ and *Lmna*$^{-/-}$ RPE cells (clone 2) were serum-starved for 24 or 48 h and then subjected to immunofluorescence stain with antibodies against Arl13b (green) and lamin A/C (red). The nuclei were stained with DAPI (blue). The representative images shown are from the cells after 48-h of serum starvation. Scale bars, 20 or 10 µm (magnified images).

D The percentage of ciliated cells as described in (C) was measured. More than 150 cells were analyzed for each group. Values (means ± SD) are from three independent experiments. Statistical significance of differences is assessed with Student's *t*-test.

 © 2020 The Authors

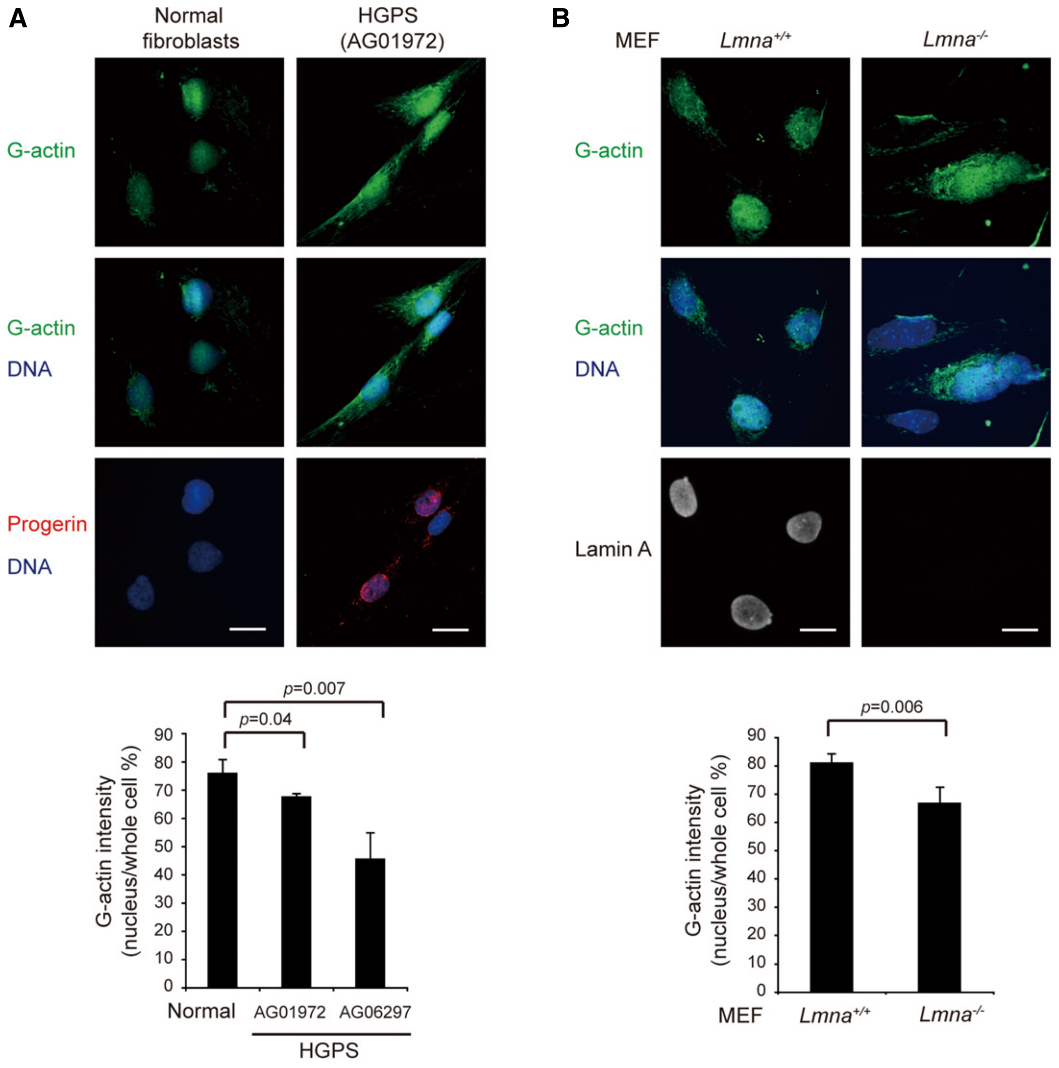

Figure EV3. The proportion of nuclear G-actin is decreased in HGPS fibroblasts and *Lmna*$^{-/-}$ MEFs.

A Normal human fibroblasts and HGPS fibroblast were stained for G-actin (green), progerin (red), and DNA (blue). Scale bars, 20 μm. The proportion of nuclear G-actin fluorescence intensity to the whole cell was measured ($n \geq 84$).

B *Lmna*$^{+/+}$ MEFs and *Lmna*$^{-/-}$ MEFs were stained for G-actin (green), lamin A (white), and DNA (blue). Scale bars, 20 μm. The proportion of nuclear G-actin fluorescence intensity to the whole cell was measured ($n \geq 92$).

Data information: Values (means ± SEM) are from three independent experiments. Statistical significance of differences is assessed with Student's *t*-test.

© 2020 The Authors

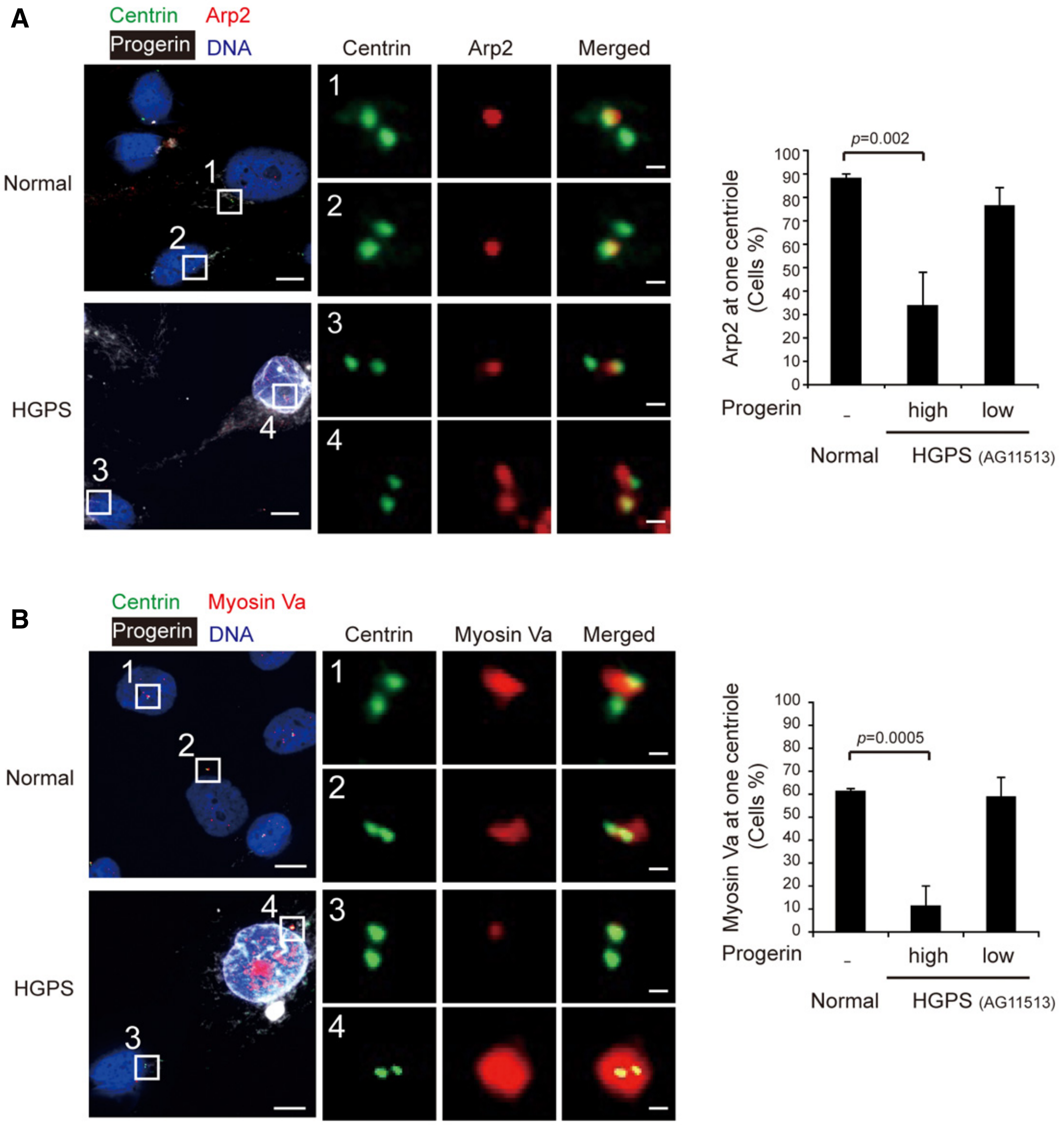

Figure EV4. HGPS fibroblasts show defective docking of Arp2 and myosin Va to the basal body.

A, B Normal human fibroblasts and HGPS fibroblasts were serum-starved for 1 h and stained for centrin (a marker for centrioles, green), progerin (white), DNA (blue), Arp2 (in panel A, red), or Myosin Va (in panel B, red). The representative images with two insets are shown. Scale bars, 10 or 0.5 μm (magnified images). Note that Arp2 and Myosin Va were found to localize exclusively at one centriole in normal fibroblasts (insets 1 and 2) and progerin-low HGPS fibroblasts (inset 3), but at both centrioles in progerin-high HGPS fibroblasts (inset 4). The graphs show the percentage of cells with Arp2 (in A, $n \geq 97$) and Myosin Va (in B, $n \geq 183$) exclusively at one centriole. Values (means ± SD) are from three independent experiments. Statistical significance of differences is assessed with Student's *t*-test.

© 2020 The Authors

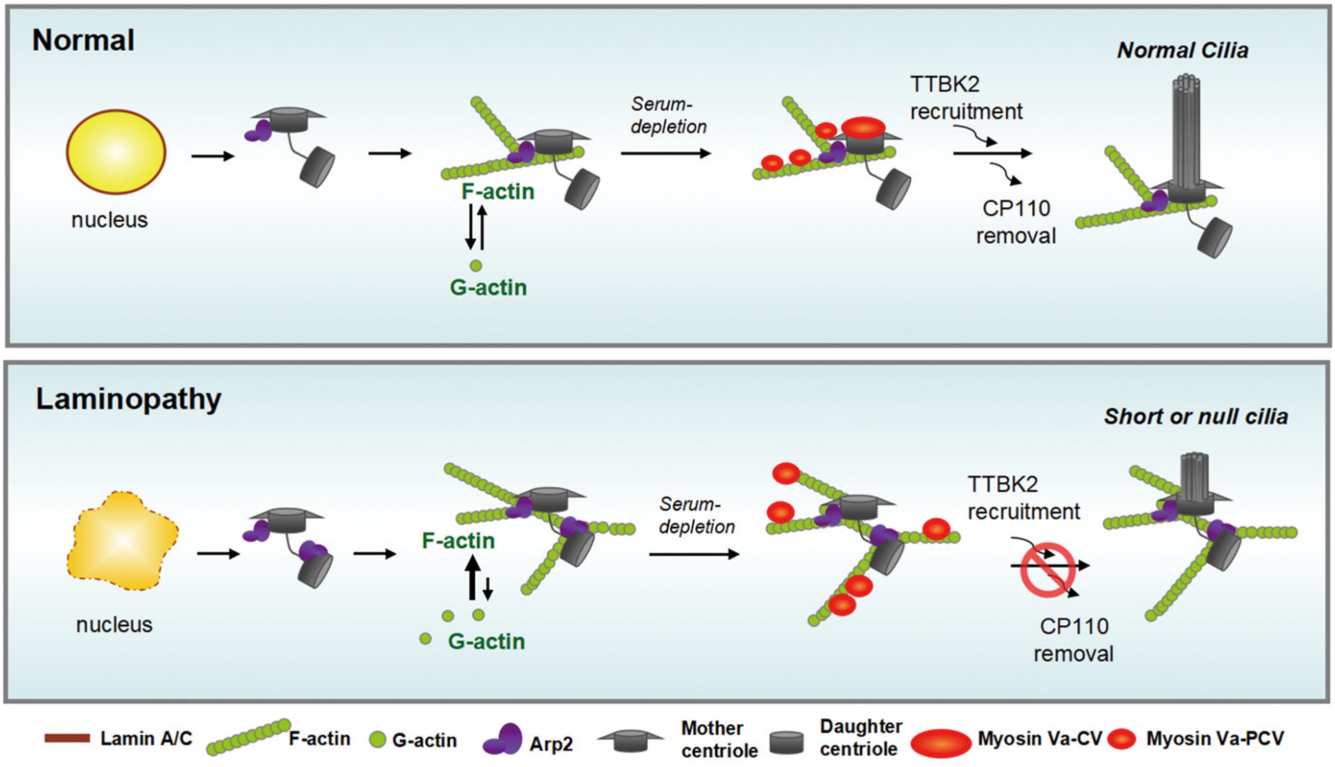

Figure EV5. A model depicting the role of lamin A/C during ciliogenesis.

In normal cells, lamin A/C-mediated integrity of the nuclear lamina allows Arp2 to localize exclusively to the mother centriole and is important for proper G-actin homeostasis between the nucleus and cytoplasm. Upon ciliogenesis, Arp2-mediated branched F-actin polymerization is occurred mainly around the mother centriole, which subsequently recruits myosin Va-PCV to the mother centriole. The formation of CV at the mother centriole is already known to be important for TTBK2-recruitment and CP110-removal, both of which are prerequisite for ciliogenesis. However, the cells with deficiency in lamin A/C (as in laminopathy) may somehow cause Arp2 localization at both centrioles, which may lead to abundant formation of branched F-actin around the basal body. The failure of myosin Va-PCV in docking to the mother centriole may cause the failure of PCV fusion into mature CV at the mother centriole, which thereby hampers the subsequent steps for ciliogenesis.

© 2020 The Authors
