# Supplementary figures and images for: Lamin A‐mediated nuclear lamina integrity is required for proper ciliogenesis
Source: EMBO Rep. 2020 Aug 19;21(10):e49680. doi: 10.15252/embr.201949680 (PMC7534621; doi:10.15252/embr.201949680)

Figure 3

A

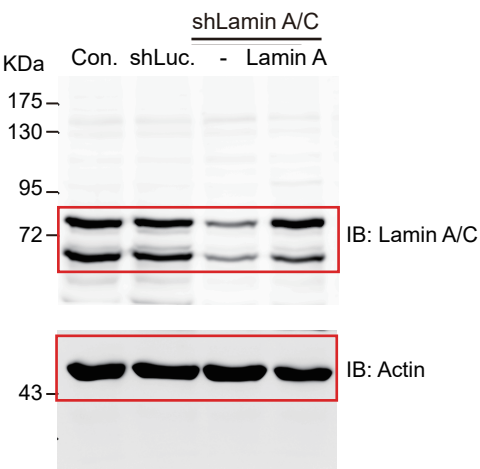

Figure 5

A

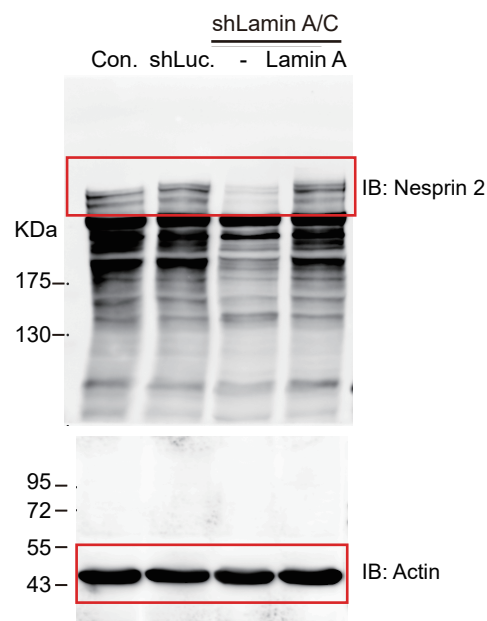

B

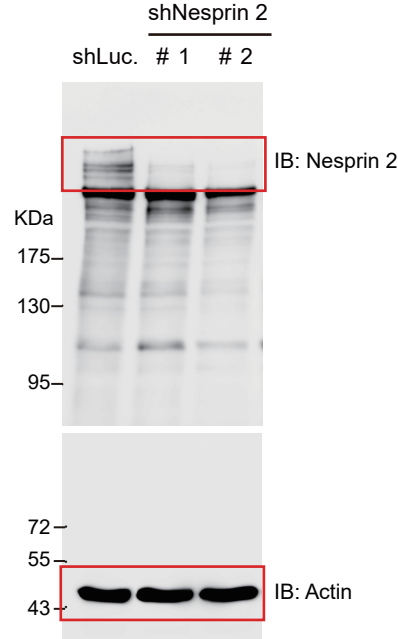

Figure 6

C

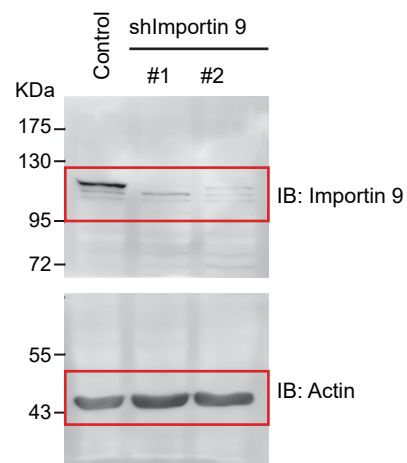

Figure S2

A

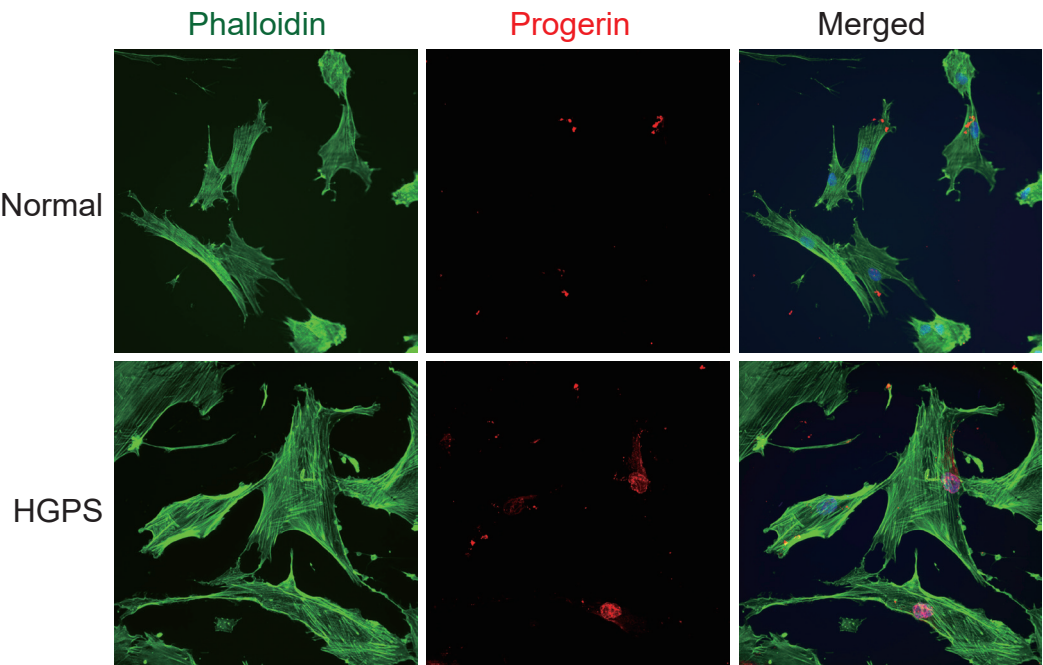

Appendix Figure S11

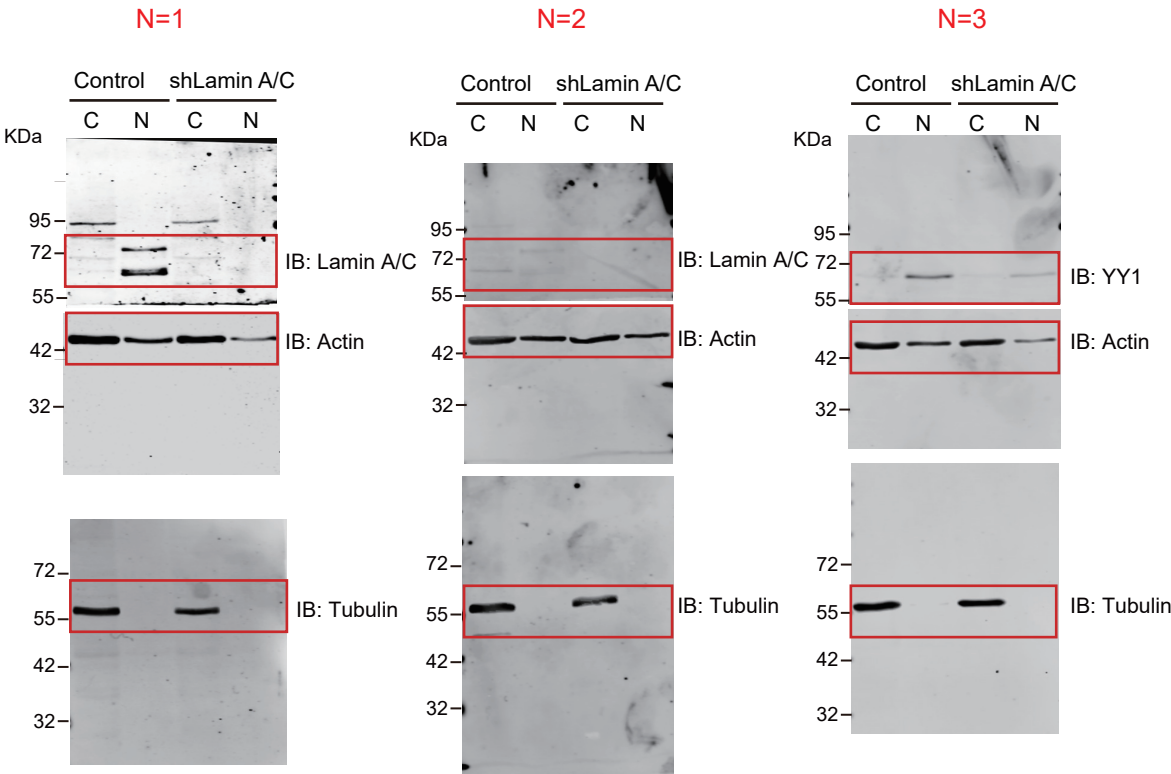

Appendix Figure S15

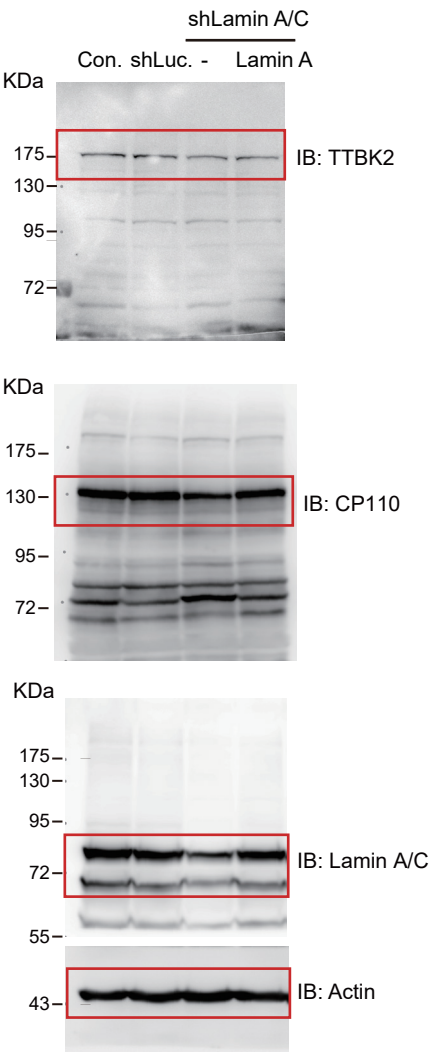

Supplement: Supplementary file 4 — Source Data for Figure 3 [file EMBR-21-e49680-s003.pdf]
